# Coordinated weather balloon solar radiation measurements during a solar eclipse

**DOI:** 10.1098/rsta.2015.0221

**Published:** 2016-09-28

**Authors:** R. G. Harrison, G. J. Marlton, P. D. Williams, K. A. Nicoll

**Affiliations:** Department of Meteorology, University of Reading, PO Box 243, Reading RG6 6BB, UK

**Keywords:** radiosonde, photodiode, radiometry

## Abstract

Solar eclipses provide a rapidly changing solar radiation environment. These changes can be studied using simple photodiode sensors, if the radiation reaching the sensors is unaffected by cloud. Transporting the sensors aloft using standard meteorological instrument packages modified to carry extra sensors, provides one promising but hitherto unexploited possibility for making solar eclipse radiation measurements. For the 20 March 2015 solar eclipse, a coordinated campaign of balloon-carried solar radiation measurements was undertaken from Reading (51.44°N, 0.94°W), Lerwick (60.15°N, 1.13°W) and Reykjavik (64.13°N, 21.90°W), straddling the path of the eclipse. The balloons reached sufficient altitude at the eclipse time for eclipse-induced variations in solar radiation and solar limb darkening to be measured above cloud. Because the sensor platforms were free to swing, techniques have been evaluated to correct the measurements for their changing orientation. In the swing-averaged technique, the mean value across a set of swings was used to approximate the radiation falling on a horizontal surface; in the swing-maximum technique, the direct beam was estimated by assuming that the maximum solar radiation during a swing occurs when the photodiode sensing surface becomes normal to the direction of the solar beam. Both approaches, essentially independent, give values that agree with theoretical expectations for the eclipse-induced radiation changes.

This article is part of the themed issue ‘Atmospheric effects of solar eclipses stimulated by the 2015 UK eclipse’.

## 1. Introduction

Solar eclipses provide an unusual opportunity to study a rapid and well-characterized change in the solar radiation entering the atmosphere. While radiation measurements related to eclipse changes have been made at the Earth’s surface [1], these can suffer from vagaries of the weather despite considerable planning, but fewer measurements have been made aloft because of the logistical difficulty and expense. A considerable attraction in using a platform aloft is that, as increasing height is achieved, the likelihood of cloud interfering with the measurements is reduced. Weather balloons, carrying meteorological instrument packages returning data by radio (radiosondes), potentially offer inexpensive platforms for such measurements. Some disadvantages, such as motion associated with the payload, limitations in weight, power and opportunities for instrument recovery, may, however, all have contributed to radiosonde platforms having been underexploited for eclipse measurements. Recent innovations in low-cost sensors have reinvigorated the utility of radiosondes as measurement platforms for parameters beyond the traditional meteorological variables. For example, a new data acquisition system has been developed to expand the science capabilities of standard commercial radiosonde systems in routine use internationally by meteorological services [2]. This system enhancement has already been used to successfully deploy a solar radiation sensor [3]. Importantly, both items are simple and inexpensive, which, as for the radiosonde itself, removes the need for them to be recovered: the instrumentation can be regarded as disposable.

For a solar eclipse, a balloon-carried solar radiation sensor brings the possibility of measuring the radiation changes away from the immediate effects of the lower atmosphere, such as the attenuating or obscuring actions of cloud, or the absorption of radiation by atmospheric constituents such as water vapour. The major eclipse-induced changes also typically tend to occur within the typical balloon flight times of 1–2 h, which provides a rare source of well-characterized variations for an *in situ* instrument. Modern meteorological balloon systems are essentially portable (e.g. allowing sampling of airborne volcanic ash in hazardous conditions [4]), but the use of an established meteorological site in the eclipse zone means that additional air traffic permissions are unlikely to be needed and that substantial items of equipment do not need to be transported. Because the typical burst height for a weather balloon carrying a standard meteorological radiosonde is at 15–20 km altitude, some of the measurements can be reliably expected to be made in air which is cloud free, hence many of the conventional climatological considerations usually applied to selecting a site for an eclipse study can be overcome.

## 2. Objectives

**Figure 1.**
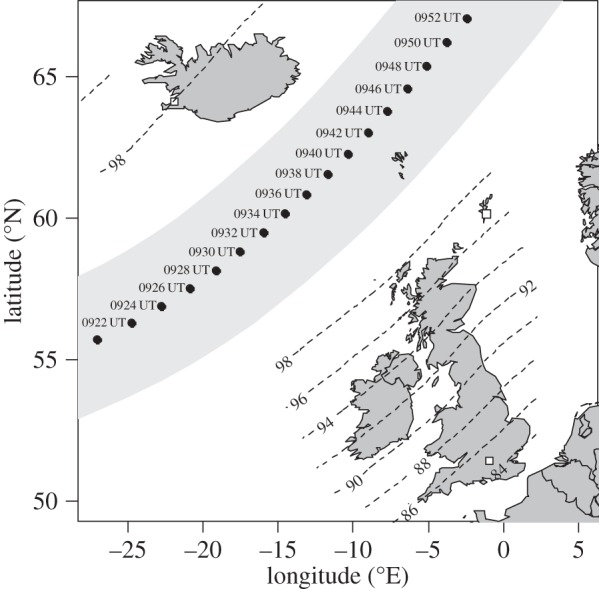
Region of totality of the solar eclipse of 20 March 2015 (grey band), with times marked. Contours of percentage obscuration are given in the regions experiencing a partial eclipse. The radiosonde launch sites at Reading (southern UK), Lerwick (Shetland) and Reykjavik (western Iceland) are marked with hollow squares.

The path of the 20 March 2015 total solar eclipse across the North Atlantic and through the Faroe Islands generated an appreciable partial eclipse in the northern UK and Iceland. This presented an opportunity for a coordinated campaign of solar radiation measurements using radiometer radiosondes, launched from the University of Reading’s Atmospheric Observatory, the UK Met Office’s Lerwick site and the Icelandic Meteorological Office facilities at Reykjavik (figure 1). The objectives of this campaign were, first, to demonstrate that the radiosonde enhancement technology could be used straightforwardly for coordinated measurements of new atmospheric variables, and, second, to investigate data processing techniques needed to retrieve quantitative radiation information from an agitated, swinging platform carrying an inexpensive sensor. Although radiosondes have been used previously in eclipse meteorology for thermodynamic measurements [5], even including multiple soundings from the same launch site [6], it is possible that the 20 March 2015 undertaking represents the first coordinated radiosonde campaign to provide widely spatially separated non-thermodynamic eclipse measurements.

## 3. Solar radiation calculations

A first consideration in configuring instrumentation for solar eclipse radiation measurements is to estimate the likely changes expected at each site. The sequence of events in a total eclipse follows from the Moon first appearing to reach the Sun (first contact) through second and third contact, between which there is the period of totality, to fourth contact when the Moon and Sun appear to emerge from each other. During the eclipse, the solar radiation is reduced from that expected for the same location and time of year, by the proportion of the solar disc’s area covered (the obscuration). Calculating the solar radiation during the eclipse can be achieved by combining the standard calculation of the daily variation in top-of-atmosphere solar radiation with a modulating function to represent the eclipse. The top-of-atmosphere solar radiation is essentially an astronomical calculation: the actual radiation in the lower atmosphere will be reduced from the top-of-atmosphere value through absorption by ozone and water vapour, which is variable.

Assuming negligible difference between the actual and mean Sun–Earth distances, the time variation in solar irradiance on a horizontal surface at the top of the atmosphere *S*_T_(*t*) is given approximately by
3.1
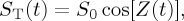
where *S*_0_ is the total solar irradiance (TSI) and *Z* is the solar zenith angle at a time *t*. For a site at latitude *ϕ* when the solar declination is *δ*, the variation in *Z* during the day is found from the hour angle *h*(*t*) as
3.2
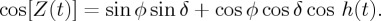
The solar irradiance variation with time at a particular position is conventionally calculated by combining (3.1) and (3.2) to give
3.3
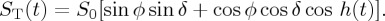
On a day with a total solar eclipse, an additional modulation function is needed to represent the effect of the eclipse. The solar irradiance can then be written as
3.4
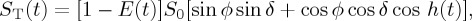
where *E*(*t*) is the *eclipse function*. In this case, the function is arranged to give the fraction of the Sun’s area covered as the eclipse progresses, with *E*(*t*)=0 at first and fourth contact. Full calculation of the eclipse function requires geocentric coordinates [7]. Instead, a simpler geometrical approximation is used [8], which represents the eclipsed Sun and Moon as two spherical bodies with an equal angular diameter at the Earth, and assumes that the solar disc is of uniform brightness with no darkening at the solar limb. These two bodies progress to overlap each other at a steady rate, with the fractional area of the solar disc remaining exposed given by
3.5
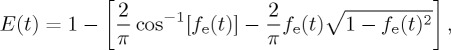
where *f*_e_(*t*) is the eclipse *magnitude*, the proportion of the Sun’s radius obscured by the Moon at a time *t*. For a total solar eclipse occurring symmetrically between first contact *t*=*t*_1_ and fourth contact *t*=*t*_4_, *f*_e_(*t*) can be defined as
3.6*a*
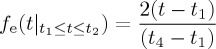
and
3.6*b*
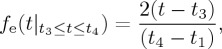
with *f*_e_=1 during totality. For a symmetrical (and non-annular) partial eclipse having a maximum obscuration *M* at *t*_*M*_, the solar radiation does not reach zero and the solar radiation variation of equation (3.4) is modified to
3.7
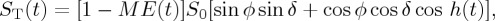
with *f*_e_(*t*) found from
3.8*a*
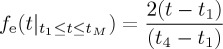
and
3.8*b*
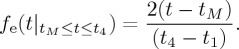
To calculate the top of atmosphere solar radiation variation on a day with an eclipse, values of *t*_1_ to *t*_4_ and *M* are required, available from eclipse tables. The other parameters required for the calculation are (i) the declination *δ*, given (in degrees) by
3.9
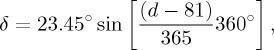
where *d* is the day of the year, (ii) the hour angle *h*, given (in degrees) by
3.10
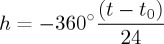
with *t* the time of day for which the solar radiation is required and *t*_0_ the time of the local solar noon in hours and (iii) the TSI *S*_0_, 1365 W m^−2^.

**Figure 2.**
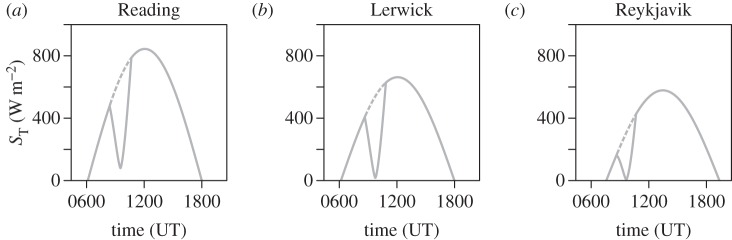
Calculated solar irradiance on a horizontal surface at the top of the atmosphere (*S*_T_) plotted against time of day (in hours UT) for 20 March 2015, with the solar eclipse included for (*a*) Reading, (*b*) Lerwick and (*c*) Reykjavik. (The dotted line marks the solar irradiance calculated for the same day without the eclipse.)

Table 1 summarizes the circumstances of the partial eclipse at Reading, Lerwick and Reykjavik, in particular the parameters *t*_1_, *t*_4_ (from which *t*_*M*_ can be found) and *M*. These have been used to calculate the variation in top of atmosphere solar radiation, *S*_T_, using equation (3.7) for each site, plotted in figure 2. The variations differ between the sites. At Reading, the eclipse begins when *S*_T_ has a larger value than at the other sites but undergoes a smaller change than at the other sites during the eclipse; at Lerwick and Reykjavik, the eclipse effects are greater, but change from smaller *S*_T_ values than at Reading.

**Table 1.** Circumstances of the 20 March 2015 solar eclipse at the three radiosonde launch sites.

site	latitude N	longitude W	eclipse start (UT)	eclipse end (UT)	magnitude
Reading	51.44	0.94	0824	1040	0.88
Lerwick	60.15	−1.13	0839	1051	0.97
Reykjavik	64.13	21.90	0838	1040	0.98

## 4. Instrumentation

The calculations in figure 2 indicate that a dynamic range of approximately 1000 W m^−2^ is needed for full measurement for the solar radiation measurement changes during the 20 March 2015 eclipse. The radiosonde radiation instrumentation described previously [2,3] was not intended for accurate radiometry, as its primary use was for detecting the radiation changes associated with cloud-to-clear-air transitions. However, in principle, the device should be capable of good radiation measurements, as the single conditioning circuitry employed a linear current to voltage converter, with the voltages recorded accurately on the radiosonde system using an analogue-to-digital converter. Furthermore, comparison at the surface against a calibrated radiometer had also shown a linear response to radiation, and part-to-part variation between the photodiode sensors used was small. The same device was therefore chosen for eclipse measurements.

For the eclipse radiation measurements, instrumentation was constructed using the previous signal conditioning circuitry [3], and the PANDORA radiosonde data acquisition system [2]. Two similar radiometers were built for each radiosonde, but with silicon photodiode sensors of slightly different spectral ranges. The typical spectral response of a silicon photodiode begins at about 200 nm and rises steadily to a maximum around 950 nm, above which it sharply loses sensitivity. In one of the balloon radiometers, a VTB8440B photodiode was used, which includes a filter to remove the response at the infrared end of the visible spectrum. In the other, a VTB8440 photodiode was used, which is an unfiltered type and has a wider spectral range. Table 2 summarizes these details. Of the two photodiodes, the filtered device approximately covers the range of visible solar radiation, with its peak spectral response at 580 nm. The unfiltered device includes the visible range, but its principal sensitivity is weighted towards the near-infrared end of its response at 920 nm, with less response in the visible region. In a subsidiary experiment, photodiodes of both kinds were compared with a calibrated radiometer to determine their response to solar radiation; a summary is given in the electronic supplementary material.

**Table 2.** Spectral response of photodiodes.

part number	λ_min_ (nm)	λ_max_ (nm)	λ_peak_ (nm)	comment
VTB8440	320	1100	920	unfiltered—broader wavelength response
VTB8440B	330	720	580	IR filter—visible wavelength response

In use for the soundings, the photodiodes were mounted on an upper horizontal surface of the plastic enclosure housing the data acquisition system, which was strapped to the radiosonde package. This added a further 130 g of payload to the 350 g mass of the radiosonde. The existing radiosonde battery was used to power the additional instrumentation. The PANDORA system was programmed to return data every 1 s over the standard UHF data telemetry, with the photodiodes sampled 64 times per second to improve the effective resolution of the 10 bit analogue-to-digital convertor employed.

## 5. Results and data processing

To increase the likelihood of the sensors being above the cloud during the time of maximum eclipse (about 0930 UT), the radiosondes were launched from each site close to 0845 UT. For an ascent rate of nominally 5 m s^−1^, this launch time was chosen to ensure that the radiosondes were above 10 km during the phase of the greatest eclipse. As well as being situated above cloud, the amount of radiative absorption from water vapour at this height is considerably reduced compared with that at the surface, and the solar radiation more closely approximates the calculated top-of-atmosphere value. The actual heights obtained from radiosondes depend, however, on the contributions of local winds, the balloons and the amount of free lift used. A further factor is that balloons can burst randomly at lower altitudes, although only rarely, for which circumstances an additional spare device was prepared as a contingency. Even so, unless such a random burst occurred at a low altitude or as part of the launch, the spare instrument was unlikely to rise sufficiently above cloud layers to give the unobstructed solar view sought. Fortunately, the contingency was not required at any of the sites.

**Figure 3.**
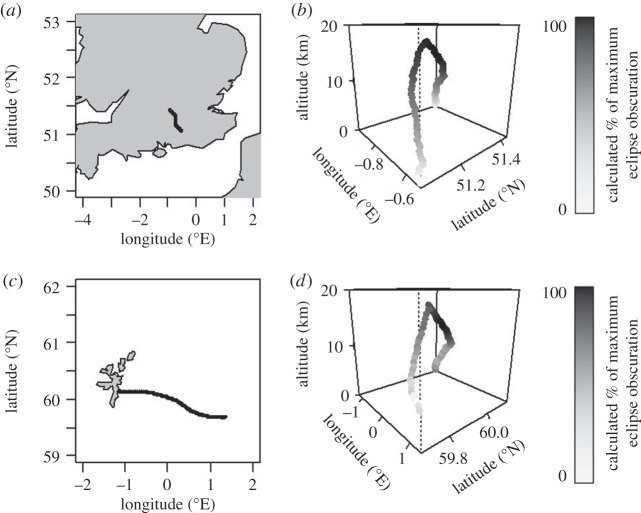
Details of balloon launches on 20 March 2015 from Reading ((*a*) flight trajectory in terms of longitude and latitude and (*b*) flight profile, with vertical height in km shown on a vertical axis) and Lerwick (as for (*a*) and (*b*), with trajectory (*c*) and profile (*d*)). On the profile plots (*b*) and (*d*), the points have been shaded according to the proportion of the eclipsed solar radiation measured at the same time, with black shading corresponding to the maximum obscuration and therefore the least radiation.

Measurements from the data acquisition system of both the unfiltered and filtered photodiode currents were merged with the standard radiosonde data of temperature, pressure, relative humidity, GPS position and flight time. The less rapidly obtained (at 2 s sampling) standard radiosonde data were linearly interpolated to give values coincident with those from the PANDORA data. Figure 3 shows the trajectories of the Lerwick and Reading balloons on 20 March 2015, derived from the standard GPS information. It is clear that the radiosondes’ altitudes during the maximum eclipse were above 10 km as planned. (Similar positional information was not available from Reykjavik, owing to a software problem, although the unfiltered photodiode data were still returned satisfactorily.)

### (a) Lerwick

**Figure 4.**
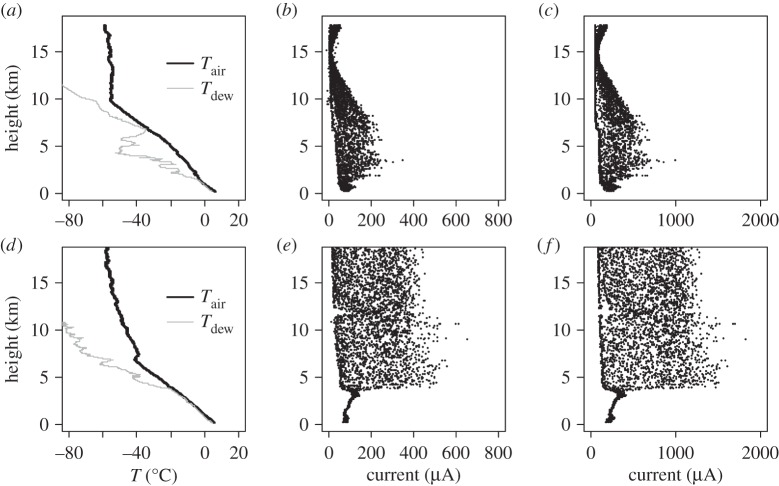
Vertical profiles of measurements obtained from the Lerwick soundings on 20 March at 0845 UT (*a*–*c*) and 1500 UT (*d*–*f*). These show: (*a*,*d*) thermodynamic data (air temperature *T*_air_ and dew point temperature *T*_dew_), and instantaneous currents measured from (*b*,*e*) the spectrally filtered and (*c*,*f*) the spectrally unfiltered photodiodes.

Figure 4 shows measured data obtained at 1 s sampling from two Lerwick solar radiation soundings on the 20 March 2015, during the eclipse launch at 0858 UT (figure 4*a*–*c*), and in the afternoon after the eclipse (figure 4*d*–*f*) launched at 1500 UT. Figure 4*a*,*d* shows the vertical profiles of measured meteorological thermodynamic variables, including the dew point temperature *T*_*dew*_. *T*_dew_ is equal to the local air temperature *T*_air_, when the air is saturated, which is a good indicator of the presence of cloud. On this criterion, low cloud is evident in both ascents, and in the lowest 2 km of the eclipse ascent, consistent with visual reports from the site. Figure 4*b*,*c*,*e*,*f* shows the raw values of instantaneous currents measured by the two photodiodes carried. These also indicate low cloud from the reduction in photodiode current in this region, implying less solar radiation. Above this, the photodiode currents become more variable, dominated by the motion of the instrument package beneath the balloon carrying the sensors in and out of the direct solar beam. For the eclipse ascent, from 10 km to the burst height at 17 km, both photodiodes show steady reduction and then recovery in output current approximately symmetrically around 15 km, which is the effect of the solar eclipse. The solar radiation profiles shown in figure 4*b*,*c* during the eclipse show markedly reduced radiation compared with the measurements made later in the day (figure 4*e*,*f*).

The variability apparent in the eclipse ascents (figure 4*b*,*c*) indicates that further data processing is needed for a comparison to be made with the calculations of *S*_T_ given in figure 2. Because the orientation of the platform is not known, an exact correction for the alignment of the sensors with respect to the incoming solar beam cannot be made. Modest swing of the platform is usual, however, and this can be used to inform assumptions about the position of the sensors. The typical period of swing was found using a Fourier transform, by regarding the photodiode measurements as a time series. This value of approximately 10 s is consistent with that of a simple pendulum for a string length of 30 m (table 3), although the actual motion may be considerably more complicated than a simple pendulum in regions of atmospheric turbulence [9].

**Table 3.** Details of instrument package deployments.

site	launch time (UT)	balloon mass (g)	string length (m)	median cycle time for swing (s)	burst height (m)
Reading	0848	200	30	9.9	17 360
Lerwick	0858	200	30	10.7	17 736
Reykjavik	0857	300	30	9.1	24 005

**Figure 5.**
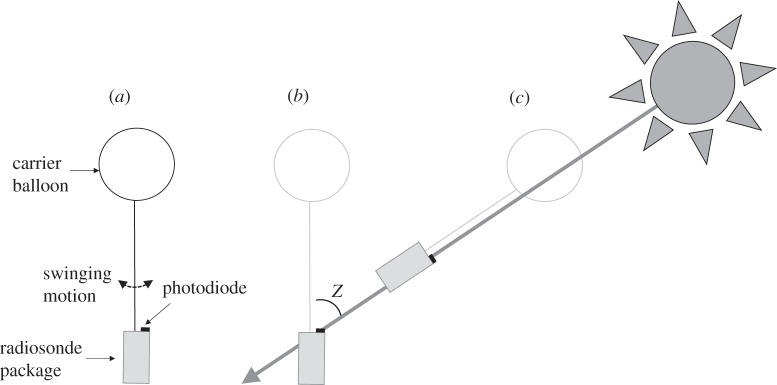
Configuration of the instrumentation used for the solar radiation measurements. (*a*) Arrangement of the radiosonde beneath its helium-filled carrier balloon, with the photodiode sensors mounted on its upward facing surface. The radiosonde package is free to swing beneath the balloon. Geometry of the Sun and photodiode when the principal solar beam is directed at a zenith angle *Z* and the photodiode sensing surface is (*b*) horizontal and (*c*) normal to the solar beam.

Figure 5 shows some possible exposure scenarios associated with the swing of the radiosonde and photodiode sensors. Figure 5*a* shows the arrangement of the instrument package beneath the balloon and position of the sensors on the upper surface of the instrument package. When hanging vertically (figure 5*b*), the position of the sensor surface is horizontal. This can then be compared with the calculation of *S*_T_ for the same time, or solar zenith angle *Z* can be used to resolve the radiation measured to that occurring on a sensing surface normal to the solar beam. The position of the sensing surface is not known. However, if the photodiode is assumed to swing symmetrically, the photodiode will be exposed horizontally at the lowest point in the swing. Its exposure to solar radiation will increase as it swings into the solar beam, and reduce as it swings in the opposite direction. Averaging measurements obtained during several swing cycles can therefore provide an estimate of the radiation obtained horizontally, and a measure of the associated variability resulting from the swinging motion.

An alternative approach is suggested in figure 5*c*. During the pendulum-like motion of the instrument package, the greatest radiation value measured will be when the sensing surface swings normal to the solar beam. Clearly, depending on the solar elevation, the swing may not be of sufficient amplitude to bring the sensing surface normal to the beam and these conditions will only be approximately obtained. However, as the solar elevations approach the local noon, the maximum value measured during the swing will provide a better approximation to the direct beam radiation at normal incidence.

**Figure 6.**
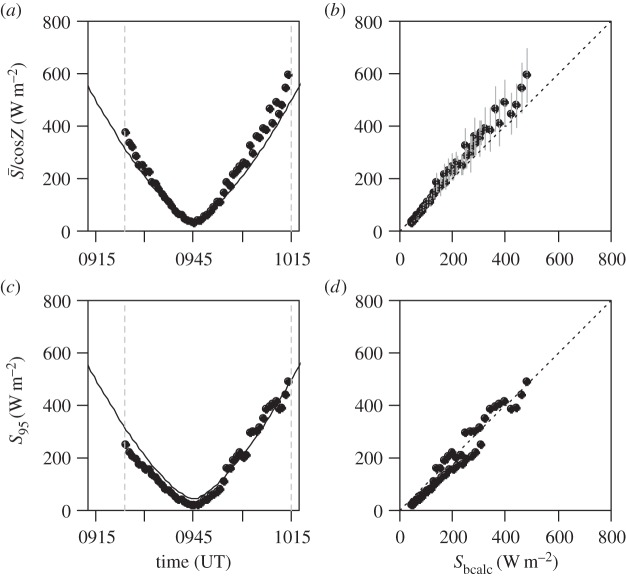
Processed data from the Lerwick 20 March 2015 ascent, for the filtered photodiode, selected for times around the eclipse. (*a*) One minute averages of measured solar radiation data corrected by the solar zenith angle *Z* (points) plotted with the calculated top of atmosphere direct solar beam *S*_bcalc_ (line). (*b*) Comparison of the calculated direct beam (*S*_bcalc_) and the data from (*a*), with a 1:1 dashed line added. (Error bars show 1.96 standard errors on the calculated mean value.) (*c*) Points show 95th percentile values chosen from 1 min of solar radiation data (*S*_95_), with *S*_bcalc_ (line), again compared directly (*d*) with *S*_bcalc_ with a 1:1 dashed line added.

Results from the two approaches of figure 5*b*,*c* are compared in figure 6, with figure 6*a*,*b* concerning the swing-averaged method and figure 6*c*,*d* concerning the swing-maximum method. For both methods, 1 min periods of the 1 s samples are calculated. This choice was informed by the median swing time of the instrument package (table 3), of approximately 10 s, which indicates that several complete cycles will usually be completed with a 1 min averaging time.

In figure 6*a*, average currents for the filtered photodiode around the eclipse time have been converted to solar radiation *S* using the linear regression found in the calibration experiment and corrected using cos *Z* at the same time to give the equivalent value normal to the solar beam. These values show a similar variation with time and similar magnitude to the calculated direct beam, *S*_bcalc_, found as *S*_T_/cos *Z*. The quantities are compared directly in figure 6*b*, with the standard error on the 1 min mean used to provide an error estimate. There is increasing deviation for the larger radiation values, but values obtained are not inconsistent with the calculated values. To evaluate the swing-maximum method, the upper 95th percentile value of each 1 min of samples has been extracted (*S*_95_) and over-plotted on the time variation of *S*_T_/cos *Z* in figure 6*c*. The upper 95th percentile was used rather than the maximum value, in case the single maximum value recorded in the 1 min period was an outlier. Again, there is agreement in shape and magnitude. Figure 6*d* compares the values from this method with *S*_bcalc_. No estimate of uncertainty is available as only one value can be obtained, but good agreement is nevertheless apparent between *S*_95_ and *S*_bcalc_.

### (b) Reading

**Figure 7.**
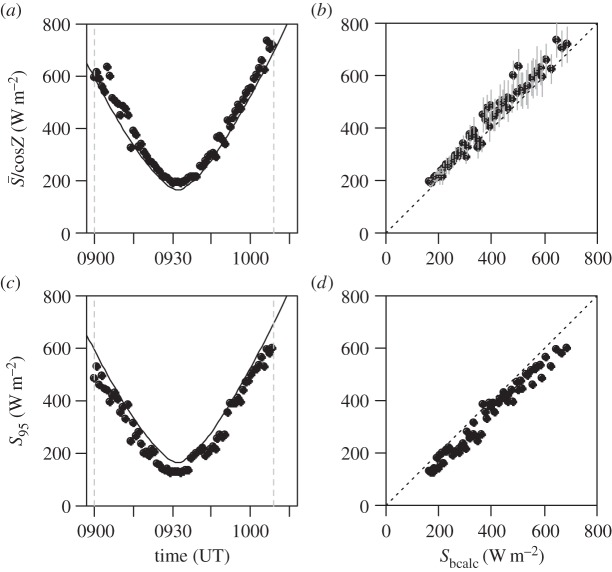
As for figure 6, but for the Reading ascent.

A similar analysis to that for the Lerwick data is used for the sounding from Reading, again for the filtered photodiode sensor. Figure 7*a*,*b* shows the results for the swing-averaged methods, and figure 7*c*,*d* shows the results for the swing-maximum method. Both methods show agreement in shape with *S*_bcalc_ values, although the swing-average method slightly overestimates the radiation and the swing-maximum method underestimates it. As mentioned above, the swing-maximum method will underestimate the radiation if the swing amplitude is insufficient to bring the sensing surface normal to the solar beam direction.

### (c) Reykjavik

Owing to software difficulties, only measurements from the unfiltered photodiode sensor were obtained from the Reykjavik sounding. While a calibration is available for these sensors from the surface experiment, the wider spectral range of the unfiltered photodiodes matches the spectrum of the visible sunlight less closely. The broader spectral range, and the peak response at 920 nm brings with it the possibility that additional sources of near-infrared radiation may contribute to the measurement, or changes in the spectral composition of the radiation as a result of the eclipse [10]. The eclipse magnitude at Reykjavik is almost total (0.98), but there is still a finite current measured by the unfiltered photodiode, when very little current was observed by the filtered photodiode during the other sites’ local eclipse maxima. This is likely to be due to additional sources of near-infrared radiation as mentioned above, or the result of a spectral shift in the remaining solar radiation to this part of the spectrum where the unfiltered photodiode is particularly sensitive. As a correction to allow the shape of the response with time to be obtained, after applying the solar radiation calibration for the filtered photodiode, the offset current at maximum eclipse has been subtracted.

**Figure 8.**
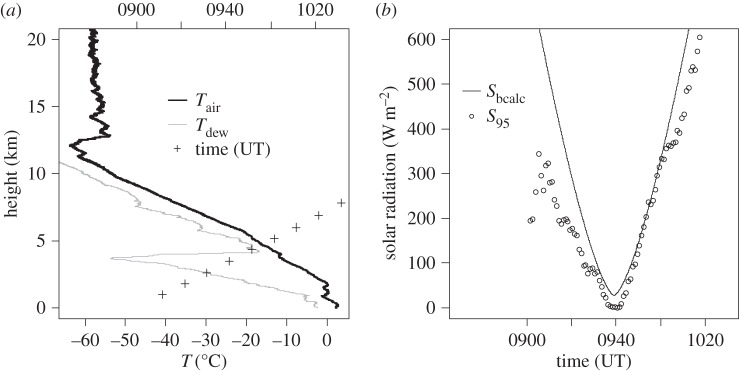
(*a*) Thermodynamic data from the Reykjavik sounding, showing profiles of air temperature (*T*_air_) and dew point temperature (*T*_dew_). Heights of the sounding against time are also plotted using + signs, with time given on the upper horizontal axis. (*b*) Measurements from the unfiltered photodiode during the eclipse (1 min mean values). Points show the 95th percentile value of solar radiation found from successive 1 min intervals containing 1 s samples (i.e. using the swing-maximum method), after subtracting an offset of 204.6 W m^−2^ at the time of the eclipse minimum. The calculated direct beam at the top of atmosphere is plotted as a line.

Figure 8 shows the data obtained from the Reykjavik sounding. Figure 8*a* shows the thermodynamic data and the height variation with time. This suggests that the instruments were in, or close to, cloud as the maximum eclipse time was approached. Figure 8*b* shows the measured radiation following the procedure described above, using the swing-maximum method. While the absolute values cannot be regarded with the same confidence as for the Lerwick and Reading ascents, because of the presence of cloud and the correction procedure necessary, there is nevertheless agreement between the *S*_bcalc_ values and the equivalent solar radiation derived from the unfiltered photodiode.

### (d) Spectral changes

**Figure 9.**
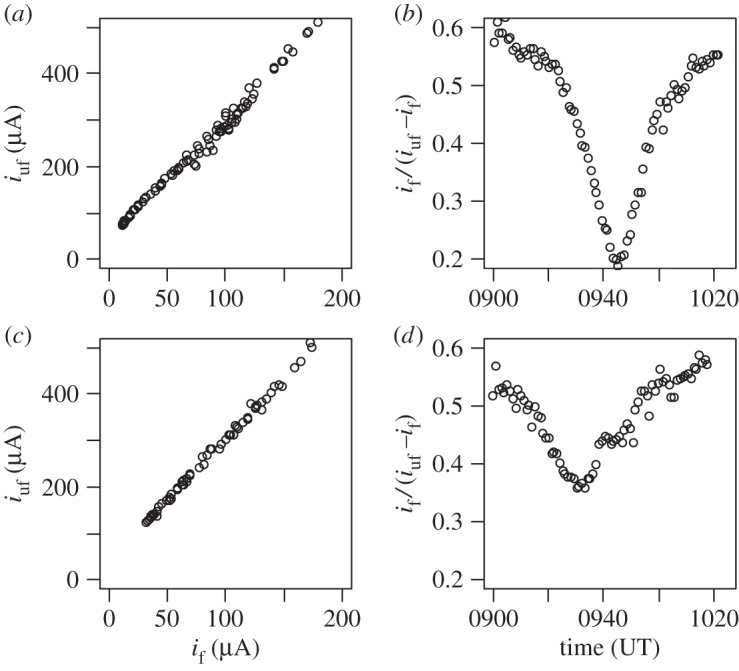
Comparison of currents from the filtered (*i*_f_) and unfiltered (*i*_uf_) photodiodes measured during the eclipse sounding for Lerwick (*a*,*b*) and Reading (*c*,*d*) using 1 min averages in each case. In (*a*,*c*), the currents obtained simultaneously from the two photodiodes on the same flight are plotted against each other; in (*b*,*d*), the ratios of the visible photodiode current (*i*_f_) to the near-infrared current, found by differencing the photodiodes (*i*_uf_−*i*_f_), are plotted against time for Lerwick and Reading.

The different spectral responses of the unfiltered and filtered (visible light) photodiodes carried on the same instrument package can be investigated by comparing their measurements during the eclipse ascent. Figure 9*a*,*c* shows the photodiode currents obtained simultaneously plotted against each other, for Lerwick and Reading, respectively. Extrapolating the filtered photodiode response to zero current (i.e. when light in the visible spectrum would be absent) shows that a finite current would nevertheless be maintained at the unfiltered photodiode, as suggested by the current measured during the Reykjavik ascent at the eclipse maximum. Further broadband spectral information can be obtained by subtracting the current measured at the filtered photodiode (wavelength range 330–720 nm) from that of the unfiltered photodiode (wavelength range 320–1100 nm), yielding the response to radiation in the range 720–1100 nm, i.e. in the near IR. Figure 9*b*,*d* shows this near IR current as a fraction of that obtained by the unfiltered photodiode, as a function of time. This ratio changed with time, and during the maximum of the eclipse dropped by about 60% at Lerwick and about 40% at Reading. This indicates, at both sites, a relative spectral shift from the visible towards the near IR range of wavelengths, with the greater relative change at the location where the eclipse was the greater.

Such a spectral change can be expected from solar limb darkening, which causes the edge of the solar disc to appear darker and cooler. More limb darkening will occur at the site with the greatest eclipse, hence the proportional change in contribution of the near IR radiation will be greater at Lerwick than Reading, as observed. Previous calculations for 11 August 1999 indicated a change of 60% at 310 nm and 30% at 1500 nm, not inconsistent with the present observations [10].

## 6. Conclusion

The coordinated use of radiosondes carrying solar radiation detectors successfully provided measurements of the solar radiation changes caused by the same solar eclipse at three spatially separated locations. Because the eclipse provides a prescribed change in solar radiation that occurs more rapidly than the typical flight time of the radiosonde, the performance of the detectors in flight can be evaluated, which is not normally possible.

In the soundings made from Reading and Lerwick, the measurements showed good agreement with a simple theoretical model of the expected changes. This encourages further use of the calculation method. Further, the agreement between model and measurements supports the use of unstabilized photodiode sensors on radiosondes for quantitative radiometry, using the swing-averaged method. In some circumstances when there is high solar elevation and appreciable swing, the swing-maximum method can provide an additional measurement, potentially independent of the swing-average method if the single swing-maximum value is removed prior to calculating the average. Finally, the simultaneous use of two photodiodes with different spectral responses on the same platform demonstrates the solar limb darkening effect. Clearly, a wider range of spectral responses could be combined in future eclipse balloon sounding experiments, or multiple narrow band sensors used simultaneously.

Other future work in this area could include performing coordinated night-time radiosonde launches during lunar eclipses. It is believed that variations in the solar radiation that is reflected by the Moon during lunar eclipses have not previously been measured using radiosondes suspended from weather balloons. Lunar eclipses differ from solar eclipses in several important respects that would need to be taken into account. First, whereas solar eclipses such as the one measured herein are visible from only a relatively small fraction of the Earth’s surface, lunar eclipses are visible across the entire night side of the Earth. Second, lunar eclipses tend to be substantially longer in duration than solar eclipses. Finally, the radiation levels and their reductions during a lunar eclipse are much weaker than for a solar eclipse, indicating that more sensitive radiometers may be required. In this respect, a partial or total lunar eclipse would be more promising than a penumbral lunar eclipse, because the reduction in reflected solar radiation is greater.

## Supplementary Material

Supplementary material

## Data accessibility

The data files for the eclipse soundings and the surface calibrations of the photodiodes are available from the University of Reading’s Research Data Archive at http://researchdata.reading.ac.uk/id/eprint/6.
